# Complete chloroplast genome sequence of *Acer nikoense* (Sapindaceae)

**DOI:** 10.1080/23802359.2020.1797574

**Published:** 2020-08-03

**Authors:** Qidi Fu, Xuedan Yu, Xinhe Xia, Yongqi Zheng, Chuanhong Zhang

**Affiliations:** State Key Laboratory of Tree Genetics and Breeding, Laboratory of Forest Silviculture and Tree Cultivation, Research Institute of Forestry, Chinese Academy of Forestry, Beijing, China

**Keywords:** *Acer nikoense*, chloroplast genome, phylogenetic analysis

## Abstract

*Acer nikoense* (Sapindaceae: Acer) is a deciduous tree, belonging to the Ser. *Grisea* of Sect. *Trifoliata*. Its complete genome sequence was obtained using genome Illumina pair-end sequencing data. It had a typical quadripartite structure with 155,952 bp in length, consisting of a large single-copy region (85,720 bp) and a small single-copy region (18,072 bp), as well as a pair of inverted repeats (26,080 bp). The total GC content was 37.9%. A total of 113 unique genes were annotated, including 30 tRNAs, 4 rRNAs, and 79 protein-coding genes. The phylogenetic analysis indicated that *A. nikoense* and *A. triflorum* were the most closely related.

*Acer nikoense* is an attractive deciduous arbor, which belongs to the Ser. *Grisea* of Sect. *Trifoliata* in the genus *Acer* of Sapindaceae. Being one of the ornamental maples, it is naturally distributed in northern Jiangxi Province, southern Anhui Province, western Hubei Province and Japan (Wu et al. 2008). According to the International Union for Conservation of Nature (IUCN), it was near threatened (NT) (Wang and Xie 2004). It is a lesser-known species, and the studies were mainly focused on propagation, chemical composition, and medicinal value (Kurimoto et al. 2016; Kim et al. 2017). Till now, the chloroplast genomes of *A. griseum* (Wang et al. 2017) and *A. triflorum* (Xia et al. 2020) which also belong to the Ser. *Grisea*, were defined, but the information of the complete chloroplast genome of *A. nikoense* is still lacking. Here, the complete cp genome of *A. nikoense* was sequenced and assembled to clarify its taxonomic status and lay a foundation for further study on population genetics.

Healthy fresh leaves of *A. nikoense* were collected from a wild individual maple from the Ningbo City, Zhejiang Province, China (N29°49′4′′, E121°33′3′′). The voucher specimen used in this study was deposited in the Laboratory of Forest Silviculture and Tree Cultivation, Research Institute of Forestry, Chinese Academy of Forestry in Beijing, China (Voucher specimen: ACNIK-ZJNB2018-01). Total genome DNA was extracted by the plant genomic DNA extraction kit (DP350) (Tiangen biotech Inc., Beijing, China) and then sequenced using the Illumina Hiseq platform (Huitong biotechnology Inc., Shenzhen, China). The de novo assembly and genome annotation were respectively performed using SPAdes v3.9.0 (Bankevich et al. 2012) and DOGMA (Wyman et al. 2004). Finally, the chloroplast genome map was drawn by OGDRAW (Lohse et al. 2013).

Being similar with most other angiosperms, *A. nikoense* had a typical quadripartite structure with 155,952 bp in length, consisting of a large single-copy region (LSC: 85,720 bp), a small single-copy region (SSC: 18,072 bp), as well as a pair of inverted repeats (IRa and IRb: 26,080 bp). The total GC content of this circular DNA molecule was 37.9%. As a result of annotation, a total of 136 genes were annotated, of which 113 are unique genes, including 30 tRNAs, four rRNAs, and 79 protein-coding genes. The complete chloroplast genome characteristics of *A. nikoense* were similar to that of *A. triflorum* (Xia et al. 2020).

Phylogenetic relationships were reconstructed using maximum-likelihood method through MEGA v 7.0.14 (Kumar et al. 2016) (Figure 1), based on the chloroplast genome of *A. nikoense* and the genomes of 23 other species downloaded from NCBI Organelle Genome Database. All sequences were aligned using MAFFT (Nakamura et al. 2018). The results clearly indicated that *A. nikoense* and *A. triflorum* were most closely related, followed by *A. griseum*, which was inconsistent with the conclusion of Wei (2019), which showed the closest relationship between *A. nikoense* and *A. griseum* by SSR markers. In addition, the genus *Acer* had the closest relationship with *Dipteronia* and were clustered into monophyly by 100% bootstrap value, which has in common with the result of Xia et al. (2020). Overall, the complete chloroplast genome of *A. nikoense* obtained in this study offered abundant genomic information for the future study on the phylogeny of *Acer* L.

**Figure 1.**
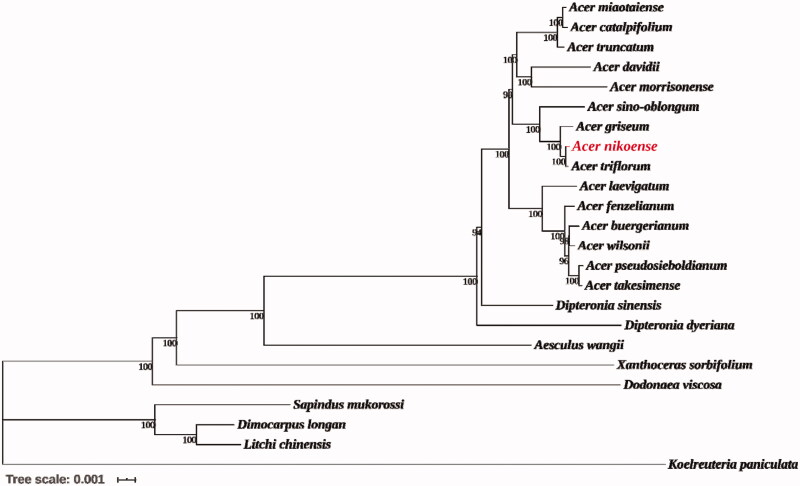
Phylogenetic tree reconstruction of 24 species using maximum-likelihood (ML) based on the complete chloroplast genome sequences of *A. nikoense* and other 23 species. There are the bootstrap support values from 1000 replicates given at each node. Their accession numbers are as follows: *Acer buergerianum*: NC_034744; *Acer catalpifolium*: NC_041080; *Acer davidii*: NC_030331; *Acer fenzelianum*: NC_045527; *Acer griseum*: NC_034346; *Acer pseudosieboldianum*: NC_046487; *Acer laevigatum*: NC_042443; *Acer miaotaiense*: NC_030343; *Acer morrisonense*: NC_029371; *Acer sino-oblongum*: NC_040106; *Acer takesimense*: NC_046488; *Acer triflorum*: MN602455; *Acer truncatum*: NC_037211; *Acer wilsonii*: NC_040988; *Aesculus wangi*i: NC_035955; *Dimocarpus longan*: NC_037447; *Dipteronia dyeriana*: NC_031899; *Dipteronia sinensis*: NC_029338; *Dodonaea viscosa*: NC_036099; *Litchi chinensis*: NC_035238; *Koelreuteria paniculata*: NC_037176; *Sapindus mukorossi*: NC_025554; *Xanthoceras sorbifolium*: NC_037448.

## Data Availability

The data that support the findings of this study are openly available in GenBank of NCBI at https://www.ncbi.nlm.nih.gov/, reference number MT216763.
